# The National Medical Union (Official)

**Published:** 1914-06-13

**Authors:** Robert Carswell, E. Arthur Dorrell

**Affiliations:** Hon. Secretaries; Hon. Secretaries


### THE NATIONAL MEDICAL UNION (Official).

Offices: 346 Strand. Tel.: City 8444.

As the present arrangements for the weekly publication of National Medical Union news have terminated with the conclusion of the work of the Provisional Council, only official notices and reports will be distributed by the Union to members through The Hospital, until the Press Committee of the permanent Council is formed to undertake the work of collecting and editing material for insertion.

Copies of the report of the Provisional Council as published in last week's Hospital have been reprinted and distributed to local associations in the form of a prospectus of the Union. It is requested that these be used for the purpose of obtaining new members for the Union before the date of the general meeting. After this date the prospectus will be re-issued for general use in a similar form, embodying the conclusions arrived at by the meeting. Additional copies may be obtained on application to the central office.

Local associations are requested to do their best to ensure a particularly good attendance of members at the dinner, which promises to be an important social function for all non-panel medical men who are united in a common hostility to the medical provisions of the National Insurance Acts, who have the welfare of the medical profession at heart, and who are in sympathy with the aims and objects of the Union. We are requested to state that the notice concerning the dinner has been altered to the effect that evening dress will be optional.

Robert Carswell,
E. Arthur Dorrell,
Hon. Secretaries.

